# Evolving treatment strategies for myeloma

**DOI:** 10.1038/sj.bjc.6602341

**Published:** 2005-01-18

**Authors:** G J Morgan, F E Davies

**Affiliations:** 1 Royal Marsden Hospital and Institute of Cancer Research, Downs Road, Surrey SM2 5PT, UK

**Keywords:** myeloma, novel treatments, thalidomide, ImiDs, proteasome inhibitors, targeted treatment

## Abstract

In this article, we will discuss how treatment strategies for myeloma have evolved and outline the challenges now faced following the introduction of a number of novel active agents. In particular, we will focus on how achieving a maximum response and maintaining such responses is becoming a key therapeutic strategy and how novel agents can be used to achieve this in the context of current strategies such as autologous transplantation.

Myeloma is a malignant disorder, which is characterised by an excess of abnormal bone marrow plasma cells producing a clonal paraprotein detectable in the serum and urine in the majority of patients. Common clinical sequelae include lytic bone lesions, fractures, myelosuppression and renal failure. It is a relatively common disease with approximately 2500–3000 new cases each year in the UK. The incidence increases with age, and the majority of cases occur over the age of 60 years. With effective treatment, the median survival is approximately 4 years. After years of few therapeutic advances, we find ourselves standing at the site of a marked expansion in treatment choices and the challenge now is how to test these new treatments against the best previously available.

Until recently, the basis of myeloma chemotherapy was aimed at the achievement of a disease phase called ‘plateau’ where there is an absence of overt clinical symptoms and paraprotein levels are stable. Despite this aim, it was generally accepted that the quality of life of patients in plateau phase was poor although a small percentage of patients lived for a prolonged period with a relatively good quality of life. With the advent of new agents that are able to achieve better responses and outcomes, it has become increasingly important to define the appropriate clinical settings and the therapeutic strategies with which to use them. The clinical settings are relatively easily recognised, including therapy for induction, maintenance, relapse and refractory disease. In this article, we will argue that the strategic aim with which to use novel treatments should be to maximise clinical responses. This is based on what has been learnt over the last 20 years and the clinical characteristics of a complete response (CR), a disease phase where there is a good quality of life and few ongoing symptoms. Responses obtained should be maintained for the maximum time period, which will require the development and implementation of novel antimyeloma therapies suitable for the maintenance setting. At present, the maximum number of CRs is achieved after high-dose therapy (HDT) (Attal *et al*, 1996; Davies *et al*, 2001a; Child *et al*, 2003), and the impact of new agents has to be compared to this current gold standard. In this setting, it is important to distinguish between agents that give good but transient responses and those for which overall responses may be less but which may be maintained for longer. Normally in myeloma treatment trials, it is conventional to consider the impact of a single line of treatment on overall survival (OS) as the major end point. However, it is becoming increasingly apparent that during the average disease course of a patient with myeloma, the patient will receive multiple courses of treatment, the sequencing of which may or may not be important. A good example of this is the observation of the beneficial therapeutic effect of HDT whether it is used at presentation or at relapse (Attal *et al*, 1996; Fermand *et al*, 1998; Child *et al*, 2003). Consequently, it is important to distinguish the end points of OS and progression free survival (PFS), both of which can give useful information, and also recognise that OS may be affected by subsequent treatment regimens.

## THE DEVELOPMENT OF CURRENT TREATMENT STRATEGIES

Initial myeloma trials showed that early death was, and still is, a significant problem, and addressing the causes of this is important including early diagnosis, the treatment of infection, hydration and the choice of the most appropriate chemotherapy. Melphalan, an alkylating agent, has been shown to be an effective treatment and no evidence of a superior treatment regimen emerged in early clinical trials, although it was shown in an early MRC trial that single agent cyclophosphamide was equivalent to melphalan (MRC, 1980). In the 1980s, several groups investigated the value of using combination chemotherapy compared to single agent melphalan. In the UK, the MRC Myeloma V trial compared melphalan alone to ABCM (adriamycin, 1,3-bis (2-chloroethyl)-1-nitrosourea (BCNU), cyclophosphamide and melphalan) and showed a significant benefit for the combination, including significant differences in achievement of plateau (61 *vs* 49%) and in median OS (32 *vs* 24 months) (MacLennan *et al*, 1992). However, systematic reviews of published and of individual patient data from a number of clinical trials did not show a significant advantage for other combinations (VMCP (vincristine, melphalan, cyclophosphamide and prednisone), VBAP (vincristine, BCNU, adriamycin and prednisone) and VCAP (vincristine, cyclophosphamide, adriamycin and prednisone)) (MTCG, 1998), although relatively few of these combinations included the use of an anthracycline as in the ABCM regime.

At the same time, alternative combinations, dose schedules and modes of administration of active chemotherapeutic agents were being explored. High response rates were reported with dexamethasone alone and in combination with VAD in which vincristine and adriamycin were given by a 4-day intravenous infusion together with high-dose dexamethasone (Samson *et al*, 1989). Dexamethasone was recognised as a key component in this regime, and good response rates were noted using this agent alone but such responses were not maintained long term. A major feature of this regimen is its lack of toxicity to the haematopoietic stem cell compartment, making it an ideal regimen for use for stem cell harvest before autologous transplantation. Concurrently, investigators at the Royal Marsden Hospital looked at the role of melphalan dose escalation. Doses of 140 mg m^−2^ were found to achieve high response rates, with CRs being reported in about 30% of patients, and for the first time bone healing was noted (McElwain and Powles, 1983). The development of autologous stem cell rescue allowed safe escalation of the dose of melphalan to 200 mg m^−2^ and resulted in even higher remission rates, with CR being reported in approximately 50% of patients compared to the very few CRs that were traditionally seen with standard dose treatments such as oral melphalan. As a result of these clinical investigations, a high-dose treatment strategy emerged, consisting of VAD initially, to induce disease response, at which stage haemopoietic stem cells are collected. These cells are then used to support high-dose melphalan, which consolidates the responses obtained. Analysis of the effect of response after HDT suggested that patients achieving a CR had a better PFS and OS than those who did not (Barlogie *et al*, 1999; Lahuerta *et al*, 2000; Davies *et al*, 2001a).

It became important, at this stage, to understand how the intensive treatments aimed at achieving maximum response rates compared to standard dose chemotherapies. The first of a series of randomised trials was carried out by the Intergroupe Francophone du Myelome (IFM) (Attal *et al*, 1996). In an intention to treat analysis, they demonstrated a significant advantage for patients in the intensive arm in terms of response rate, response duration and survival, with a median OS of 56 months compared with 44 months in the standard arm. The MRC Myeloma VII trial addressed the same question, comparing ABCM with C-VAMP (cyclophosphamide, vincristine, adriamycin, methylprednisolone) followed by high-dose melphalan (Child *et al*, 2003). A total of 400 patients aged less than 65 years were randomised. There was a significant improvement in OS of patients treated with intensive therapy compared with those treated conventionally, with a median survival of 54 months compared with 42 months. Response rates and response durations were also improved in the HDT arm. A published data meta-analysis of similar trials confirmed the beneficial therapeutic effect of the intensive treatment approach, establishing it as the standard treatment for suitable presenting patients.

Over the years, a number of maintenance strategies have been investigated in order to prolong responses post-HDT and improve quality of life. Interferon has been used in this setting but has not been taken up widely on account of a number of features. It significantly impaired quality of life, was expensive, and in meta-analyses, although it was shown to be statistically significantly better than no maintenance therapy, the benefit was not clinically significant (MTCG, 2001). Dexamethasone has also been used, and in a small study looked effective but again the side-effect profile particularly in the elderly has not led to its general uptake (Berenson *et al*, 2002). The therapeutic profile of a drug useful in the maintenance setting is likely to be different from one used in the induction phase of treatment, and while efficacy is important, tolerability is crucial.

## NOVEL TREATMENTS

To date, the majority of new agents have been tested in the relapse and refractory setting in order to determine clinical efficacy and safety profiles. Many of these agents are now being moved into earlier disease stages where combination regimens based on *in vitro* synergy can be tested. VAD is the classical treatment used to achieve response before HDT. New therapies, working by alternative mechanisms, incorporated into induction regimes prior to transplant may improve the numbers of responses achieved before transplant. They could also be used after transplant as part of a strategy to maximise responses for patients who fail to achieve a CR, if they did not induce severe myelosuppression. An alternative to this approach, if the responses suggested in initial studies are achieved, is that novel combinations could be used in place of HDT. However, as HDT is recognised as an effective regimen, it is still likely to be used at some stage of the disease process. Before novel combinations can be compared with HDT in a randomised setting as part of induction therapy, the response rates and lengths of remission need to be firmly established; however, such a comparison is tempting as intensive therapy involves periods in hospital with a long period of recovery.

## THALIDOMIDE AND ITS DERIVATIVES

The recognition that thalidomide was active in myeloma was based on its antiangiogenic activity in *in vitro* models together with the unexpected description of neo-angiogenesis in the bone marrow of myeloma patients. Initial studies of single agent thalidomide in relapsed refractory disease showed a remarkable 30% response rate (Singhal *et al*, 1999). *In vitro* characterisation of its mechanism of action demonstrated a variety of effects including direct toxicity to the myeloma plasma cell, alteration in cytokine secretion, interruption of the myeloma cell stromal cell interaction and increase in T and NK cell activity (Hideshima *et al*, 2000; Davies *et al*, 2001b). Thus, thalidomide is not a classical cytotoxic providing a rationale for combining it with other treatments. The addition of thalidomide to standard regimens for older less-fit patients unsuitable for transplant options is of great clinical interest. Complete responses with MP (melphalan and prednisone) are in the order of 10%, but preliminary data using MPT (melphalan, prednisone and thalidomide) suggest that these rates can be increased to 50%, a level comparable to those achieved with HDT (Palumbo *et al*, 2003). Current questions relate to whether this improved response rate translates to improved survival and if it does, the balance of standard *vs* intensive treatment may alter and necessitate a specific randomised comparison.

Regimens have also been developed including combinations of thalidomide and dexamethasone with or without cyclophosphamide (Rajkumar *et al*, 2002; Cavenagh and Oakervee, 2003; Weber *et al*, 2003). These combinations represent an oral version of VAD and have the potential to improve significantly pretransplant responses, at the same time removing the need for an indwelling catheter. An ongoing phase III study, MRC myeloma IX, is comparing CTD (cyclophosphamide, thalidomide and dexamethasone) with CVAD (cyclophosphamide, vincristine, adriamycin and dexamethasone). However, it seems highly likely that these new regimes will be more effective and be associated with fewer side effects than VAD and will become the new standard induction therapy prior to HDT. Intensification of these regimes further by the addition of other agents has been useful for refractory patients. DT-PACE (dexamethasone, thalidamide, cisplatin, adriamycin, cyclophosphamide, etoposide) has been developed for patients who have not responded to standard treatments and are candidates for HDT. This regimen is effective and able to rescue significant numbers of patients who can then proceed to HDT (Lee CK *et al*, 2003). Whereas other maintenance strategies have not been widely taken up, thalidomide maintenance for patients who have achieved a response is appealing because of its multiple modes of action, especially its immunomodulatory effect. It is currently being explored in this setting, in particular looking at its side-effect profile. A UKMF phase II study of 84 patients receiving thalidomide maintenance post-HDT suggests that long-term treatment is possible at low doses (<200 mg) but that peripheral neuropathy and other toxicities can affect its continued use (Feyler *et al*, 2003). In spite of this down side, at a recent meeting in Torino, preliminary results from the IFM suggest an improvement in PFS when it is used following HDT. It is not unreasonable to suggest that similar effects would be seen after treatment with oral standard dose chemotherapy.

For this reason, thalidomide derivatives offer an interesting alternative because of their favourable side-effect profiles. A number of such derivatives of thalidomide including Revlimid™ and Actimid™ have been developed, which potentially offer the opportunity of a decrease in side effects (phocomelia, thrombosis, peripheral neuropathy) with an increase in antimyeloma activity. The immunomodulatory drugs (IMiDs) are 50 000 times more potent at inhibiting TNF secretion, more potent inducers of T-cell proliferation with IFN and IL-2 secretion, and inhibitors of IL-1B and IL-6 secretion, and in *in vitro* studies demonstrate an increased myeloma cell kill (Hideshima *et al*, 2000). Initial phase I and II studies of Revlimid™ and Actimid™ are extremely encouraging with response rates between 38 and 58% with CRs in the order of 10% and no significant somnolence, constipation or neuropathy (Richardson *et al*, 2002, 2003a; Streetly *et al*, 2003). A phase III randomised trial is ongoing.

## BORTEZOMIB AND PROTEASOME INHIBITION

Bortezomib (Velcade™) is a boron-containing molecule, which reversibly inhibits the proteasome. The 26S proteasome is a large multi-subunit protein, which is present in all eukaryotic cells and functions to degrade proteins targeted to it by ubiquitination. Ubiquitinated proteins enter at one end of the proteasome and are degraded to their individual peptides, which are shed from the far end of its barrel-like structure. It consequently has a critical role in maintaining intracellular homeostasis allowing complex intracellular signalling events to take place, which are essential for the control of cell cycle progression, transcription and apoptosis, as well as mediating inter-cell signalling events such as those leading to chemotaxis, angiogenesis and adhesion (Adams, 2004). Proteasome inhibition with bortezomib can induce apoptosis even in myeloma cell lines resistant to conventional chemotherapy, suggesting that it works by a distinct mechanism not affected by the drug resistance mechanisms leading to alkylator and steroid resistance. One central mechanism by which Bortezomib functions in myeloma is likely to be via inhibition of the breakdown of I*κ*B and consequently stabilisation of the NF-*κ*B complex. This prevents NF-*κ*B translocation to the nucleus with consequent inactivation of multiple downstream pathways. In addition, other molecules stabilised by proteasome inhibition include p53, p21 and p27, and one possible mechanism of apoptosis is the simultaneous accumulation of cell signalling and cell cycle regulatory molecules. Bortezomib decreases the adhesion of the myeloma plasma cell to stromal cells, which increases sensitivity to apoptosis, as well as interrupts prosurvival paracrine and autocrine cytokine loops in the bone marrow microenvironment mediated by IL-6, IGF1, VEGF and TNF*α* (Hideshima *et al*, 2001).

A phase II study of 202 patients with relapsed refractory myeloma demonstrated that 35% of these heavily pretreated patients achieved a response to treatment and 10% had a CR or near CR (Richardson *et al*, 2003b). These are truly remarkable response rates for this late stage of the disease, suggesting that even greater response rates would be seen earlier in the natural history of the disease. The median survival was 16 months with a median duration of response of 12 months. Response to treatment was associated with improvement in a range of clinical parameters and quality of life. A phase III trial has recently been closed early due to the improved efficacy in disease free survival of bortezomib over dexamethasone. Much like with thalidomide, bortezomib combinations are being developed based on *in vitro* data (Ma *et al*, 2003). There is evidence for an additive effect with dexamethasone, and this is supported by data from the phase II trial in which patients who failed to respond to bortezomib alone showed evidence of a response when dexamethasone was added. *In vitro*, at nontherapeutic doses, it has been possible to sensitise cell lines to the cytotoxic effects of melphalan, doxorubicin and mitoxantrone (Mitsiades *et al*, 2003). Ongoing clinical trials are using bortezomib in combination with other agents, including melphalan, thalidomide and cyclophosphamide (Yang *et al*, 2003; Zangari *et al*, 2003). These combinations may be particularly effective for refractory disease and could potentially achieve greater response rates than current high-dose treatment strategies and could be the subject of randomised comparisons. It remains an open question as to the stability of remissions induced by such combinations, and the potential role of thalidomide/revlimid™ maintenance needs to be considered.

## TARGETED TREATMENTS ON THE HORIZON

Targeted treatment is not a new concept and has in fact underpinned strategies for treating cancer since their inception. However, novel targeted approaches rely upon the identification of a specific molecular target and the design of specific small molecules to inhibit them. The activity of proteasome inhibition in myeloma highlights the importance of protein checkpoints and also points towards the importance of the unfolded protein response in plasma cells (Lee AH *et al*, 2003). Indeed protein synthesis may be the Achilles heel of the myeloma cell as it clearly distinguishes plasma cells from many other cells within the body. In this respect, it is not surprising that preclinical work evaluating inhibitors of molecular chaperones using HSP90 inhibitors has been shown to be effective in myeloma models. The study of multistep models of cancer progression has suggested that in addition to genetic changes such as translocation and loss of heterozygosity, heritable epigenetic effects such as methylation of CpG islands and modulation of chromatin structure via acetylation of histone tails can lead to loss of expression of key genes and disease progression. The key feature of these changes is that they are amenable to therapeutic manipulation, which can result in upregulation of gene expression associated with normalisation of the cellular phenotype. Agents in clinical trial in myeloma include the demethylating agents 5-azacytadine and decytabine and the histone deacetylase inhibitors SAHA and LAQ.

As our understanding of the pathogenesis of myeloma improves, the heterogeneity in outcome is becoming increasingly obvious. Current prognostic factors such as B2M and more recently the International Staging System (ISS) although effective are not biologically based (Greipp *et al*, 2003). It is widely anticipated that expression microarrays will identify patterns of gene expression, which can be used to define outcome, identify targets and direct treatment decisions. Perhaps the best examples of how to target treatment rely upon defining a specific molecular lesion known to be important. In myeloma, approximately 15% of patients harbour the t(4;14), which deregulates the tyrosine kinase FGFR3 and can be targeted using specific inhibitors. Inhibiting this is effective in cell line models, but proof of concept in humans has not yet been completed (Trudel *et al*, 2004). If this is achieved, it will argue for the molecular characterisation of myeloma before selecting a treatment, and would suggest that therapeutic approaches should be tested in trials for specific molecular subtypes. In order to bring this approach to fruition, it is important that we invest in the full characterisation of the myeloma genome.

## CONCLUSION

In the future, descriptions of myeloma currently characterised by the statement ‘myeloma is an incurable disease’ will be replaced by statements such as ‘myeloma is a highly treatable condition, which is associated with prolonged survival and good quality of life if the correct treatment decisions are made’. While this is an accurate description of the current clinical situation, this message has not yet fully reached the majority of physicians who treat myeloma. Achieving the full advantage of these new therapeutic options will rely upon making these approaches available to all patients diagnosed with myeloma at an early stage in the natural history of their disease. Making this possible will involve education for general practitioners and hospital physicians alike. In addition, once patients are diagnosed, equitable access to novel therapeutics in an environment where the haemato-oncologist using the drugs has adequate experience in their use is also important and provides a strong argument for the establishment of specialist clinics and centres that treat adequate numbers of myeloma and can advise on the delivery of treatment. The expansion of new therapeutics means that we have to develop systems to evaluate and rapidly introduce agents shown to be effective into the clinical arena. This demands a full interaction between the pharmaceutical industry, clinicians and the regulatory authorities.
